# An Exploratory Study on Virtual Reality Technology for Fall Prevention in Older Adults with Mild Cognitive Impairment

**DOI:** 10.3390/s25103123

**Published:** 2025-05-15

**Authors:** Wing Keung Ip, Jeffrey Soar, Kenneth Fong, Szu-Yao Wang, Christina James

**Affiliations:** 1 School of Society, Community & Health, University of Bedfordshire, Luton LU1 3JU, UK; 2 School of Business, University of Southern Queensland, Toowoomba, QLD 4350, Australia; 3 Department of Rehabilitation Sciences, The Hong Kong Polytechnic University, Hong Kong, China; 4 School of Nursing, Midwifery & Paramedicine, Australian Catholic University, Banyo, QLD 4014, Australia

**Keywords:** virtual reality (VR), cognitive-motor training, older adults, mild cognitive impairment (MCI), fall risks, fall prevention, cave automatic virtual environment (CAVE)

## Abstract

**Highlights:**

**What are the main findings?**

- The Virtual Reality (VR) training program has positive training effects on fall prevention for older adults with MCI.
- The VR technology supports a useful cognitive-motor training on fall prevention for older population.

**What is the implication of the main finding?**

- Health professions can support the creation, inclusion, and adoption of accessible sensing technologies in aged care and rehabilitation services.
- Fall prevention assisted by the VR training can provide a new training approach in preventing falls for older adults with MCI.

**Abstract:**

Introduction: Virtual Reality (VR) training has potential evidence for reducing the risks of falls of older adults with mild cognitive impairment (MCI). There are indications of a positive training effect of a cognitive-motor intervention method to improve the postural balance and cognition for safer walking. This study aimed to evaluate the training effects of VR training for reducing the risks of falls among older adults with mild cognitive impairment (MCI). Methods: An experimental design was employed to evaluate how the participants attended a full-immersive VR Cave Automatic Virtual Environment (CAVE) training program. Fifty-five participants were randomly assigned to the VR group or the control group. The VR group received 16 training sessions over 8–10 weeks, while the control group received a non-VR falls prevention program. The primary outcome assessed any falls after the study, and the secondary outcomes assessed changes in cognition and executive function, walk speed and balance performances, and the psychological factor such as fear of falling relating to the risk factors of fall. Results: The VR group showed significantly greater improvement than the control group in terms of measures of cognitive-motor performance across group and time interaction. However, there were inconsistent results in functional mobility and fall efficacy between the two groups. Conclusion: This study provides promising evidence on the VR CAVE training for reducing the risks of falls among older adults with MCI from Hong Kong. VR technology-based applications are an emerging area in current aged care and rehabilitation services.

## 1. Introduction

Preventing falls in older adults is a public health challenge that continues to attract research interest [1]. Fall is defined as an event that results in a person coming to rest inadvertently on the ground or floor or other lower level. Falls, trips, and slips can occur on one level or from a height [2]. Significantly, older adults with cognitive impairment and dementia are five times more likely to be hospitalized than older adults without cognitive impairment [3]. The literature suggests that there is a strong relationship between executive function and falls; executive dysfunction doubles the risk for future falls and increases the risk of fall injury by 40% in older adults living in the community [4]. Evidence suggests that cognitive-motor interventions, such as Virtual Reality-based training, may reduce fall risks in older adults with mild cognitive impairment and dementia [5]. However, empirical research using VR technology intervention in occupational therapy is limited [6]. The study aimed to investigate the effectiveness of Virtual Reality (VR) training as a meaningful occupation and an alternative cognitive motor training to reduce the fall risk by altering cognition, physical risk factors of falls, and the fear of falling in older adults with MCI.

VR is a human-centered technology interface that involves a real-time simulated environment and interactions through multiple sensory channels [7]. VR technology can be either semi-immersive or fully immersive. The adoption of VR CAVE training can be an innovation for the health profession. Researchers could advocate for the pilot use of an innovative training tool for preventing falls in older adults during the pandemic. Rehabilitation therapists are increasingly using assistive technology and Kinect motor sensor technology for older adults on home modifications, a safe footwear device, e.g., a fall sensor, Kinect with Xbox or Nintendo Wii, and a tele-rehab educational program for falls prevention [8]. This study became a pilot and pioneer research project using a commercially available VR CAVE application on fall prevention for older adults with cognitive impairment. This study could support an emerging area for the adoption of accessible technology in future rehabilitation and aged care services [9].

VR CAVE intervention is an innovative approach in health professions, growing rapidly, especially in rehabilitation and aged care [6,10]. The VR CAVE game activities provide simulated virtual environments, which engage the users with the holographic stimulation of virtual experience [11,12]. VR training may be a useful adjunct to falls prevention approaches in health-related applications. Supported by European projects such as the iStoppFalls, Farseeing, and PreventIT projects, this research explores using technology to improve older adults’ physical health and functioning [13]. For instance, the iStoppFalls project used exergames to reduce the risk of falls in older adults. These projects provided an essential background and supporting evidence for fall prevention through the adoption of technology. Exercise programs and interprofessional fall prevention programs are recommended as a useful fall prevention intervention strategy: clinicians such as physiotherapists and occupational therapists can use a professional-led fall prevention program in aged care services [6,14,15]. Due to the COVID-19 outbreak between 2019 and 2022, the human-guided fall prevention program had been severely restricted or reduced in community aged care services. This could lead to an adverse effect on older adults’ physical mobility and cognitive decline but also induce loneliness, boredom, and isolation. The new development of VR CAVE training can be an effective training platform to provide a holographic training environment for older adults in the post-pandemic era. The use of VR technology is becoming more affordable and can be easy to operate as compared to therapist-lead exercise. It offers potential as a safe and interactive (alternative) approach to fall prevention strategy in the adoption of future healthcare applications.

There have been reports that wearing a head-mounted device may induce discomfort and agitation in older people [16]. To minimize the impact of motion sickness or dizziness when using VR technology applications, the VR CAVE technology is designed to be more age friendly as 3D stereoscopic glasses are more convenient and comfortable for older adults than normal VR headsets. This research uses a fully immersive VR Cave Automatic Virtual Environment (CAVE) associated with sensing technology. The CAVE technology asks the participants to wear stereoscopic glasses, which enable them to see 3D graphics and images so that they can walk all the way around the objects in a simulated scenario and obtain a full view and a better understanding of exactly how those objects look and what the real-time environment is like. The adoption of VR CAVE training can be an innovation for the health profession. In addition, there exists a knowledge gap in developing useful clinical practice applications for fall prevention by using VR technology to reduce the fall risks among older adults with mild cognitive impairment [17]. Therefore, the study’s objective is to evaluate the potential training effects of VR CAVE training for fall prevention in older adults with MCI. Health professionals could consider the adoption of accessible VR CAVE technology in future healthcare practice.

This study highlights the significance of VR CAVE technology in aged care, demonstrating its benefits for health outcomes, training, and social engagement through immersive, interactive experiences [18,19]. Unlike conventional VR headsets, VR CAVE’s room-scale design with 3D stereoscopic eyewear and motion sensors offers an age-friendly solution for older adults [20,21]. While VR CAVE has been used in fields like medicine and engineering, its application in aged care—particularly for fall prevention in older adults with mild cognitive impairment—remains underexplored. As the first known study to investigate VR CAVE for this purpose, this research could drive further investment and innovation in aging-related health technology. Additionally, by aligning with global fall prevention initiatives, VR CAVE presents a promising tool for aged care stakeholders to enhance service delivery through human-computer interaction.

## 2. Methods

This study was a quasi-experimental design with three measurements from pre-test, post-test and 3 months follow up. This study adopted a convenience sampling method as a feasible alternative at the COVID-19 pandemic.

The stakeholders of community aged care facilities offered two hybrid modes including telecare and limited center-based social support services. With consent given from all stakeholders, the research was conducted in the university VR training center or the community aged care service centers.

### 2.1. Participants

The sample size of this study was two hundred and three. All potential participants were involved in screening sessions for fall risks assessments co-organized by the research team and three aged care facilities. They were referred to by the staff through a promotion leaflet of the VR fall prevention research program held between June 2021 and November 2021. They provided informed consent to participate in this study.

### 2.2. Inclusion Criteria

The target population included the service members from three service settings in Hong Kong. The inclusion criteria were (1) aged 65 years to 85 years inclusive; (2) had a history of a fall within the past two years; (3) were living in a non-residential age housing; (4) were at risk of mild cognitive impairment, which was assessed using the validated screening tool of Hong Kong Montreal Cognitive assessment test (HK-MoCA score ≤ 25), indicating a risk of mild cognitive impairment or early dementia; and (5) were able to commute independently. These criteria must be satisfied prior to enrolment procedures in community aged care facilities.

### 2.3. Exclusion Criteria

The exclusion criteria were medically unfit and had physical complaints, such as dizziness, motion sickness, epilepsy, Parkinson’s disease, and mobility impairments, and mental health challenges.

### 2.4. Intervention

The intervention program was called the VirCube VR for Rehab program, designed by a local information technology company. The VR company provided continuous technical and maintenance services in the research period. The ownership of the program belonged to the Department of Rehabilitation Sciences of the Hong Kong Polytechnic university. The researcher of this study declared no conflict of interest with the VR company; all research coordination, communication, and operations were restricted to the VR research office of the university.

The university VR CAVE research center installed the VirCube VR for Rehab program for research purposes. To match the aims of this study, cognitive-motor training programs were chosen for the intervention program. The VR game modules for this study included fire emergency handling, outdoor walking, balancing game activities, and community daily practices. The training modules involved dual task components; the participant was expected to train up his/her cognitive motor performances in an intensive VR technology-based training program. VR group participants received two sessions per week, with 16 training sessions in total. Each session consisted of three to four training modules. Each module took 10–15 min to complete with a short break before the next module. The VR training session was about 45 min and was monitored by the research team. The VirCube VR for Rehab program had detailed instruction and training protocol (Supplementary Materials File S1).

The control group did not receive any VR training in the community aged care centers. They received ordinary center-based social care service in community aged care centers. Some services resumed center-based health check service and social support services during pandemic. The control group participants were invited to take pre-test, post-test, and 3 months follow up measurements in the same period of VR group (T1, T2, and T3 intervals) in respective service centers.

### 2.5. Procedure

The VirCube VR for Rehab program and all VR CAVE facilities were built in a university VR research center. The VR participants could commute independently to receive the 16-session VR training. In every VR session, all participants were supervised by the research team. They could stop the training at any time to avoid overloading during the repeated VR training. After completion of the VR session, the research team would collect the participants’ feedback and provide a debriefing session. Pictures A to G display the VR wearing devices, VR CAVE physical set up, and cognitive-motor VR games (Supplementary Materials File S1). The VR game instruction and training protocol are documented in Supplementary Materials File S2.

### 2.6. Data Collection and Outcome Measures

The research participants gave consent to provide demographic information including personal, past falls history and other health information, such as global cognitive status and past medical history. The measurement of fall risk focuses on different risk factors of falls [22]. Fall efficacy included the concern of the fear of falling [23,24].

### 2.7. Cognitive Measures

The level of cognitive function was screened using the Hong Kong Montreal Cognitive Assessment (HK-MoCA version). This validated assessment tool covered four domains, including attention, executive functions/language, orientation, and memory. The total score ranged from 0 to 30, with a higher score indicating better cognitive function. This screening tool had good validity in detecting people with mild cognitive impairment [25]. A score of 25 or below indicated a higher risk of cognitive impairment or decline. Testing the executive function of research participants was one of the indicators to detect the change in cognitive level after VR intervention. Two executive function tests were chosen, such as the Trail Making Tests (TMT-A and TMT-B), for measuring the changes in executive function performance [26].

### 2.8. Physical Measures

The intrinsic risk factors of falls were mainly assessed through physical balance and stability, as well as walk speed, for the participants in three intervals. Three validated assessment tools, including the Berg Balance Scale (BBS), 6 min walk test (6 MWT), and Time Up and Go test (TUG), were used to measure the physical balance and mobility of the older adults at risk of falling [27].

The physical balance and level of stability were assessed using BBS. It consisted of 14 motor tasks; each scale ranged from 0 (unable) to 4 (independent). The higher the score, the greater the physical balance and stability. The BBS scale was a sensitive tool for older adults in an ageing population [28]. For instance, scores below 45 indicated individuals with a greater risk of falling. A score below 51 with a history of falls indicated a predictive risk of falls.

Another standardized fall risk assessment tool for older adults was the TUG. The participants were asked to get up from a chair, walk 3 m, turn around, walk back to the chair, and sit down, and the time taken was recorded [29]. Those participants scored the TUG (below 12 s) indicating a lower risk of fall. The 6 MWT indicated the participants’ walking speed and physical level of tolerance: the longer the walking distance, the stronger their physical condition.

### 2.9. Psychological Consideration

Fear of falling was measured using the Fall Efficacy International Scale (FES-I). This scale was a 16-item self-rated questionnaire in a Chinese version. The participants rated a score for the level of fall concern while engaging in daily occupations. It was a 4-point Likert scale with 1 as low concern and 4 as high concern of falling. It indicated that the higher the score, the higher the fear of falling. The FES-I (Chinese version) was divided into three levels, 16–19 indicating low concern of falling, a score of 20–27 indicating moderate concern, and a score of 28–64 indicating high concern [24]. This study chose the validated FES-I tool to assess the level of fall concerns between the intervention group and the control group.

### 2.10. Statistical Analysis

The primary outcome recorded the number of falls after the study, and the secondary outcomes compared the changes in measurable variables at the three points (Time 1, Time 2, and Time 3). The statistical method used SPSS version 27 and a *p* value set ≤0.05. Table 1 shows the participants’ information, including the baseline demographic characteristics of participants, including age, gender, educational level, living and cognitive status, history of fall, and past health history, such as chronic pain, osteoporosis, and fracture.

To compare the physical health outcomes between the two groups across time occasions, the repeated measures analysis of variance (ANOVA) was used for data analysis. ANOVA was selected to compare means across the two groups (e.g., intervention vs. control, pre-test vs. post-test) while controlling for Type I error, as it is robust for testing hypotheses about differences between three or more conditions [30]. ANOVA assumptions were rigorously tested. Where violations occurred (e.g., cognitive MoCA score distribution at baseline), log transformation successfully normalized data (Shapiro–Wilk *p* = 0.12 post-transformation). All analyses used two-tailed tests (α = 0.05) with effect sizes and 95% CIs. For significant main/interaction effects, Bonferroni-adjusted pairwise tests were run with effect sizes reported as η^2^p (ANOVA) and Cohen’s d (pairwise). This study design involved repeated measures and between-group comparisons, making ANOVA the appropriate choice for analyzing the effects of VR CAVE training on outcomes such as balance, cognition, or fall risk. The independent variable was a time factor; the dependent variables included cognitive and physical functions. When any variable between the two groups showed a significant difference in baseline measurement, a comparison of covariance was applied. However, there was missing data found in the study, and an intention-to-treat analysis was performed according to a last observation carried forward method.

## 3. Results

### 3.1. Demographic Data

**Figure 1:**
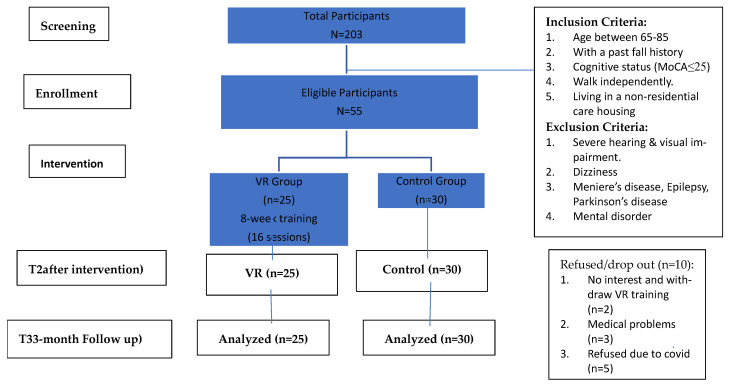
Consort diagram of this study.

Fifty-five participants were considered eligible and were given formal consent to enroll in the study (Figure 1); the meanage was 74.84%. About 89.1% of the participants were female, half of the participants had an education level of secondary or above, and about one-third of the participants were living alone in the community (non-residential aged care facility). They were classified as having a higher risk of mild cognitive impairment or dementia (mean score HK-MoCA = 21.22), and they had a higher rate of repeated falls within 12 months (50.9%). They had reported chronic pain (38.2%) and a history of fracture (29.1%) due to a fall.

Ten eligible enrolled participants dropped out unexpectedly due to medical reasons (*n* = 3) and loss of interest (*n* = 2) in the VR group; some (*n* = 5) in the control group refused to continue due to spreading anxiety. In total, 10 participants dropped out throughout the study; the refusal rate in the VR group (*n* = 2) was less than that of the control group (*n* = 5). Finally, 84.62% (*n* = 55) participants were analyzed to investigate the relationship between Virtual Reality technology-based training and the risk factors of fall.

As shown in Table 1, there were no significant differences between the two groups in any of the demographic information. All participants were female in the intervention group; they had a higher education level (60% with secondary level) than the control group (46.7%). The intervention group had a higher incidence rate (53.3%) of fall history (<12 months), the history of fracture (40%), and chronic pain (44%). The control group had a higher incidence rate (50%) of history of osteoporosis than the intervention group (35.3%).

### 3.2. Demographic Data (Health Outcomes)

As shown in Table 2, there were no significant differences (*p* > 0.05) between the two groups in executive function (TMT-A and TMT-B), balance (BBS,) and 6-min walk (6 MWT) tests. Three other outcome measures, including cognition (HK-MoCA), functional status (TUG), and fall efficacy (FESI), showed a significant difference (*p* < 0.05) at baseline measurement. The HK-MoCA mean score (VR M = 22.68; control M = 20.00) of cognitive status in the two groups indicated a higher risk of cognitive decline, such as mild cognitive impairment or dementia. The cut-off score of the HK-MoCA screening tool was 22, indicating a higher risk of cognitive impairment, which is recommended for further medical investigation [25,31]. The functional mobility (TUG, mean score = 15.01) of the control group indicated a higher risk of falls. Regarding the fall efficacy international scale (FES-I), the two groups indicated a high concern of falling (high concern of fall when FES-I > 28). Some variables in the two groups were not identical at the baseline measurement.

Importantly, the fall incident in the VR group (*n* = 2) indicated about 50% lesser rate of fall than the control group (*n* = 5) after the study. Hospital admission of participants (*n* = 3) was reported in the control group only.

The study’s secondary outcomes were illustrated in the table of multivariate and univariate analyses of dependent variables between groups and time effects (Table 3). There were significant differences in cognition (HK-MoCA, *p* = 0.008), executive function (TMT-A, *p* = 0.38, TMT-B, *p* = 0.006), balance level (BBS, *p* = 0.032), and walk speed (6 MWT, *p* = 0.001) between the two groups at pre-test, post-test, and follow-up. These outcomes indicated greater improvement effects between groups and times in the intervention group. However, there were no significant differences in functional mobility (TUG, *p* = 0.938) and fall efficacy level (FES-I, *p* = 0.148) between groups and occasions. The effect of executive function (TMT-B, *p* = 0.172) showed an inconsistent result within the VR group in the follow-up. The intervention group (TMT-B, mean difference = −10.28 s) showed a faster time than the control group (TMT-B, mean difference = +30.18 s). For the balanced outcome measure, the mean score (BBS, at post-test = 52.84 and follow-up = 53.21) indicated less predictive risk of falls (BBS score > 51) in the intervention group. The mean score of the Time Up and Go Test (TUG) of the intervention group at post-test (9.27 s) and follow-up (8.46 s) below 12 s indicated a lesser risk of falls compared with the control group. The HK-MoCA means scores of the intervention group at post-test (25.72) and follow-up (25.96) indicating a lesser risk of mild cognitive impairment or decline than the control group. Although the fall efficacy showed no significant difference between the two groups, the intervention group showed greater improvement in the mean scores of FES-I (mean score = 39.00 at post-test and mean score = 33.48 at follow up), but the mean score indicated no significant decrease in fear concern of falling (FES-I > 28) after intervention. Figures S2–S8 show the changes of different variables over time between the two groups (Supplementary Materials File S3)

## 4. Discussion

### 4.1. Overview

This study demonstrates significant improvement in cognitive-motor functions among older adults with MCI following VR CAVE training (VirCube VR). The results support the promising evidence of greater improvement in physical outcomes on the VR group after training. The VR CAVE technology application proved to be an alternative training method on falls prevention program for older adults with MCI and dementia [32,33,34].

### 4.2. Full Immersive VR CAVE Training on Falls Prevention

It is an exploratory study using the innovative application of the VR CAVE program, focusing on reducing the risks of falls among Chinese older adults with MCI in Hong Kong. The research design selects simulated VR games relating to the aims of the research project, designing and modifying a stimulated falls prevention program in the VR group. The findings align with recent reviews supporting VR training’s potential effects on fall prevention. The holographic VR games were specially redesigned and beneficial for people with old age [35,36]. Unexpectedly, the research design counters shortcomings in limited workforce resources, in addition to the VR system and facilities accessibility during the COVID-19 pandemic in Hong Kong.

This study suggests an optimal VR CAVE training protocol of twice-weekly sessions over 8 weeks, with each VR session lasting less than 45 min and 16 training sessions in total [32,35,37]. For the VR CAVE program, each participant was advised to take a break to avoid mental fatigue in between every VR game. The overall feedback of VR participants was very encouraging and rewarding. Few participants (16% drop out rate) reported quitting the VR training for health and personal reasons. The VR motion sickness was minimized with 3D stereoscopic eyewear, which is a handy VR CAVE design. This study allowed flexibility for participants to complete the 16 sessions for longer than 8 weeks (about 2 months) because of the unexpected circumstances during the pandemic in Hong Kong. In the end, a high completion rate (100%) of the participants was achieved in the study. The participants also expressed extremely high satisfaction with improving their physical health outcomes and reducing the fear of falling after the innovative VR CAVE program. The study did not cover the qualitative data to measure the participant-observational evaluation. Advisably, it could be a good suggestion to consider a focus group or a participant survey on their intentions and perceptions of using VR technology in the future.

### 4.3. Training Effects of VR Training for Fall Prevention

Our findings confirm that VR CAVE training effectively stimulates cognitive-motor functions. They align with prior evidence supporting VR’s role in fall prevention for older adults with MCI [32,38]. The literature review indicated that cognitive impairment was associated with a higher risk of falls, and a poor postural balance was found to further increase this risk [39,40,41]. People with MCI showed a higher risk of falls than community-dwelling older adults [42]. Beyond that, the VR CAVE training proved highly interactive and stimulating, fostering participant motivation, cohesiveness, and compliance with the falls prevention program.

However, functional mobility and fear of falling show inconsistent improvements between groups. At baseline (T1), TUG and FES-I scores differed significantly. VR participants showed higher functional mobility level and less fear concern of falling. The intervention effect of the VR group was not statistically significant due to a potential selection bias. Statistically, the mean scores of the fear concern of falling of the two groups were similar after the VRT intervention. The VR group had shown significant improvement than the control group on fall efficacy.

However, the VR CAVE program’s primary design focuses on cognitive-motor intervention (dual-task component), which may not be sufficient to address the fear of falling. The 16-item self-rated questionnaire (FES-I) is a subjective assessment scale and targeted for the general ageing population, with items mainly focused on daily living tasks. Participants with cognitive decline found it difficult to interpret and understand each question, particularly under so many restrictions for living in the community during the pandemic period in Hong Kong. The FES-I international scale may not be the best standardized assessment tool to assess the fear of falling, specifically for older adults with MCI. Overall, the results may be impacted by selection bias, the suitability of assessment tools, and other environmental constraints. Because the training program was conducted in The Hong Kong Polytechnic University, the participants had to spend longer time on commuting which was indeed a tougher physical demand to undertake for the training sessions. Comparatively, those participants with lower physical mobility and higher fear of falling would become more reluctant and have difficulties opting for the VR group. In general, the VR participants showed better functional mobility (TUG) than the control group.

### 4.4. Challenges and Limitations

Compared with other VR research [34,43,44], the recruitment of participants for this study is challenging. The sample size of the study is small, but the dropout rate (15.38%) seems comparatively low. Convenience sampling could lead to potential bias and affect the reliability of the findings in the older population [45]. The VR group achieved a 100% attendance rate for VR training. The participants actively engaged in the VR CAVE training with positive perception and acceptance using the application of the VR CAVE program. The other benefits of this study is good social and peer support, as well as great enjoyment in learning VR technology, among the participants in the study. Certainly, the positive effect of face-to-face interaction and interactive learning could promote being mentally and physically active and eliminate social isolation and boredom during the pandemic [10,34]. The findings of this study are identical to these observations, indicating that the VR CAVE training is effective and has a positive training effect. Future research should investigate participant acceptance and perceptions of fully immersive VR interventions.

This study has limitations, including its non-randomized design, which may affect validity. Logistical constraints during the pandemic limited participant recruitment and data collection. However, this approach was both practical and representative, as participating social care centers (e.g., The Salvation Army and St. James’ Settlement) serve diverse older adults reflective of Hong Kong’s aging population. To further reduce bias, recruitment was blinded: center staff (unaware of study hypotheses) screened volunteers against predefined criteria (e.g., age ≥ 65, history of fall), and the research team finalized enrolment without direct screening involvement. Secondly, the improvement of cognitive and executive function tests as repeated measured using MoCA and TMTA/B results could be affected by repeated learning [31]. Thirdly, the group size between the intervention group and the control group is not identical, and the VR participants showed higher motivation and engagement to take part in the research. These factors could contribute to a potential bias effect. Fourthly, the VR CAVE intervention has limited functions and merely focuses on intrinsic factors of reduction of fall risks because it is a tailor-made dual-task virtual simulated training program. According to the World Health Organization (WHO), falls prevention was complex to manage, and a multifactorial program was used to reduce the multiple risks of falls because of their complexity [46,47].

Finally, the challenges and demands for participants in the VR group are higher than the control group. The research team consumed workforce resources and faced difficulties in tackling research coordination, including the participants’ availability, facility arrangement and other technical issues. The data collection of the follow-up was heavily suspended due to a complete lockdown in Hong Kong between January 2022 and May 2022. The Hong Kong Polytechnic university and the community social service centers suspended all research projects and daily services in Hong Kong. The 3-month follow-up work in the two groups was delayed. The process of data collection might have adversely affected such as the higher dropout rate and unforeseeable factors because some participants refused to continue taking the study during this period. As an alternative, the research team adopted a special arrangement to maintain contact with the participants by phone call and other social means, e.g., WhatsApp group. Thus, this research adopted the intention-to-treat method for data analysis.

## 5. Conclusions

This study provides evidence supporting the potential training effects of VR training for fall prevention in older adults with MCI by VR CAVE training (VirCube VR). This program demonstrates potential as a fall prevention tool in aged care and rehabilitation services. This intervention could become an innovative and alternative training approach in future healthcare practice. Health professions (e.g., physiotherapists and occupational therapists) could integrate VR into rehabilitation programs. However, the demand for technological support when using VR applications is essential for older populations. Future studies should adopt larger sample sizes and randomized control trial (RCT) designs to strengthen the generalizability of VR CAVE training for fall prevention in older adults with MCI.

## Figures and Tables

**Table 1:** Baseline characteristics of the participants (*n* = 55).

Characteristic	Intervention (*n* = 25)	Control *(n* = 30)	* *p* Value
Age (years), M (SD)	71.96 (5.11)	77.23 (4.59)	0.067
Gender *n* (%)			0.180
Male		20%	
Female	100%	80%	
Education Level (years), *n* (%)			0.179
Primary or below	40%	53.3%	
	60%	46.7%	
Living Status, *n* (%)			0.208
Alone	24%	40%	
With family	76%	60%	
History of Fall, *n* (%)			0.218
<12 months	53.6%	33%	
>12 months	46.4%	67%	
History of Chronic Pain, *n* (%)			0.418
Yes	44%	33%	
No	56%	67%	
History of Fracture, *n* (%)			0.104
Yes	40%	20%	
No	60%	80%	
History of Osteoporosis, *n* (%)			0.311
Yes	35.3%	50%	
No.	64.7%	50%	

Pearson’s chi-square (two-sided) was used for categorical data. * *p* < 0.05.

**Table 2:** Baseline outcome measures of participants (***n*** = 55).

Outcomes, M ^a^ + SD ^b^				
Variables	All (*N* = 55)	Intervention (*n* = 25)	Control (*n* = 30)	*p* Value < 0.05
Cognition: HK-MoCA ^c^	21.22	22.68 (0.69)	20.00 (0.63)	0.006
Executive Function				
Trail Making Test A (TMT-A)	67.91	65.84 (8.45)	69.63 (7.71)	0.742
Trail Making Test B (TMT-B)	112.64	104.28 (75.44)	119.61 (69.60)	0.421
Time Up and Go Test (TUG)	13.41	11.48 (0.93)	15.01 (0.85)	0.007
Berg Balance Scale (BBS)	50.71	50.71 (0.98)	50.70 (0.90)	0.988
6-min walk test (6 MWT)	310.51	318.52 (12.18)	303.84 (12.03)	0.415
Fall Efficacy Scale International (FES-I)	40.11	43.04 (2.31)	37.16 (2.10)	0.043

M ^a^: Mean. SD ^b^: Standard Deviation. HK-MoCA ^c^: Hong Kong–Montreal Cognitive Assessment.

**Table 3:** Comparison of outcome measures between and within groups (intervention group *n* = 25, control group *n* = 30).

Outcome Measures	Group	Pre-Test	M + SD Post-Test	Follow Up	Multivariate	Univariate Within Group	* *p* < 0.05 Between Group
Cognition (HK-MoCA)	VR Control	22.68 (0.69) 20.00 (0.63)	25.72 (0.79) 20.97 (0.72)	25.96 (0.81) 21.2 (0.74)	0.008 **	0.000 **	0.000 **
Executive Function TMT-A TMT-B	VR Control VR Control	65.85 (8.45) 69.63 (7.14) 104.28 (13.96) 119.61 (12.74)	47.29 (6.93) 61.68 (6.32) 83.16 (14.49) 109.83 (13.22)	42.94 (6.24) 64.52 (6.07) 72.88 (15.41) 140.01 (14.66)	0.038 * 0.006 **	0.000 ** 0.172	0.038 * 0.041 *
Balance Level BBS	VR Control	50.72 (0.98) 50.70 (0.90)	52.84 (1.65) 47.33 (1.15)	53.21 (1.16) 50.13 (1.05)	0.032 *	0.311	0.047 *
Walk Speed 6 MWT	VR Control	318.51 (13.18) 303.84 (12.03)	365.10 (12.82) 298.42 (11.07)	373.68 (13.02) 305.19 (11.89)	0.001 **	0.002 **	0.002 **
Functional Mobility TUG	VR Control	11.48 (0.93) 15.01 (0.85)	9.27 (0.56) 12.61 (0.51)	8.46 (0.71) 12.07 (0.66)	0.938	0.000 **	0.000 **
Fear of Fall (FES-I)	VR Control	43.64 (2.31) 37.16 (2.10)	39.00 (2.35) 38.80 (2.15)	33.48 (2.01) 33.53 (1.84)	0.148	0.001 **	0.299

Outcome measure: A better outcome is represented by an increase in HK-MoCA, BBS, and 6 MWT; a decrease in TMT-A&TMT-B, TUG, and fall efficacy. M = mean. SD = Standard Deviation. * *p* value: significant at <0.05 level of significance. ** *p* <0.005.

## Data Availability

All authors support sharing the research data and are available from the corresponding author upon request.

## Supplementary Materials

The following supporting information can be downloaded at: https://www.mdpi.com/article/10.3390/s25103123/s1, File S1: VR CAVE set up; File S2: VirCube VR training protocol; File S3: Figures S2–S8 change in cognitive-motor performances.
