# Supplementary material for: An Exploratory Study on Virtual Reality Technology for Fall Prevention in Older Adults with Mild Cognitive Impairment
Source: Sensors (Basel). 2025 May 15;25(10):3123. doi: 10.3390/s25103123 (PMC12115703; doi:10.3390/s25103123)

Supplementary materials

File S1:

Pictures A to G displayed the VR wearing devices, CAVE VR physical set up and cognitive motor VR activities.

Picture A. VR headset (3D stereoscopic eyewear)	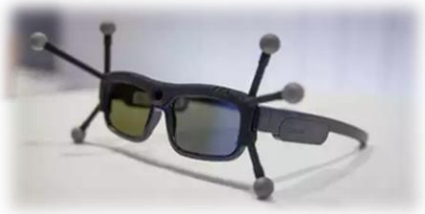
Picture B. CAVE VR Room Design<br>Specifications:<br>Room size: 2meter x 2meter<br>4 overhead projectors	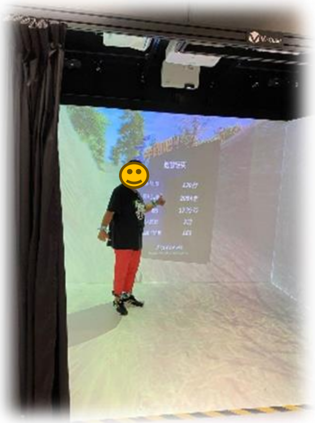
Picture C. sensors attached to 4 limbs.	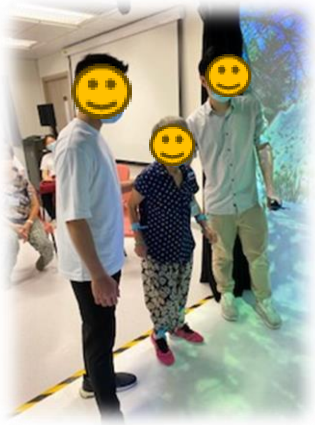

Picture D: Physical VR exercise (soccer)	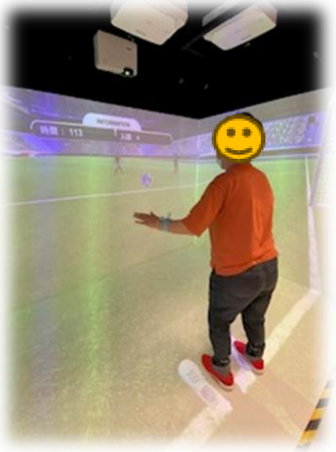
Picture E: Physical VR exercise (jogging)	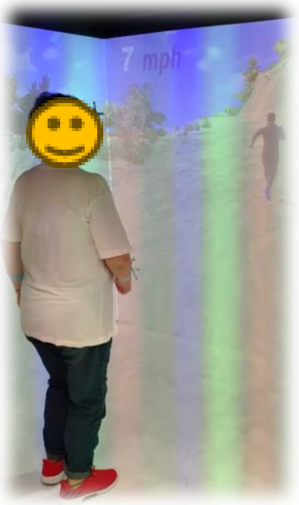
Picture F. Cognitive motor VR game (IADL-community shopping)	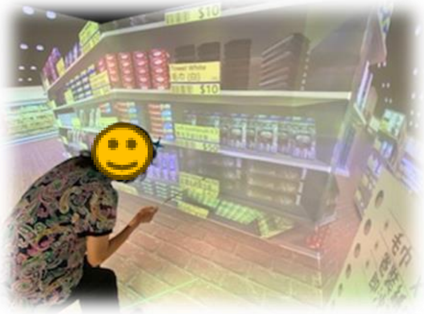

Picture G. Executive function VR game (emergency handling)	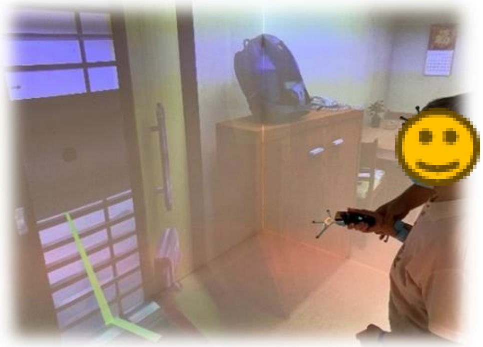

File S2:

VirCube VR Game Training Protocol (2021 version)

VR Game 1: **Emergency handling**

Purpose: Cognitive training - executive function (Problem-solving skill)

Grading: N/A

Device: 3D stereoscopic eyewear, hand controller, hand trackers

Fire drill exercise 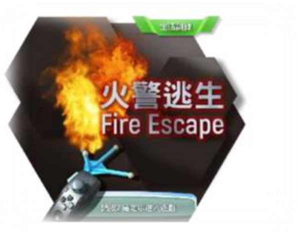	Sequencing:<br>1. When fire alarm on, participant moves tracker to point out and touch three important things (key, mobile & wet towel) for evacuation.<br>2. Participant walks to the front door and use tracker to unlock the door (balance and hand-eye coordination)<br>3. Participant uses tracker to select different escape routes (judgement)<br>4. Participant chooses either route 1(lift) or route 2(up/downstairs) to get out of fire scene safely (decision making)<br>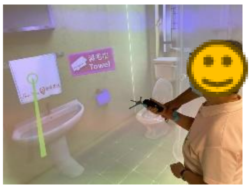 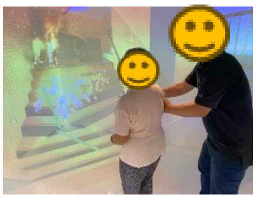 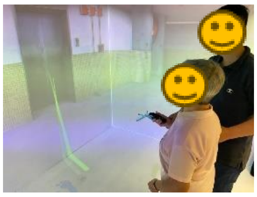

VR Game 2: **Community Living Skill Practice**

Purpose: Cognitive training – executive function (classification & calculation)

Grading: 3 levels (Level 1- free choice; Level 2: shopping items- 2-3; Level 3: shopping items- 3-5)

Device: 3D stereoscopic eyewear, hand controller

Shopping orientation	Sequencing & Grading:
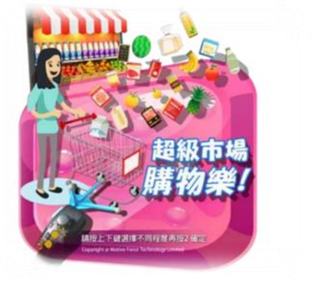	1. Participant uses hand controller selects a level of activity<br>2. Participant remembers a shopping list (memory skill)<br>3. Participant goes to the selected shopping regions (making decision)<br>4. Participant chooses and pick up the correct no. of items (classification & counting)<br>5. Participant checks and goes to cashier for payment (calculation)<br>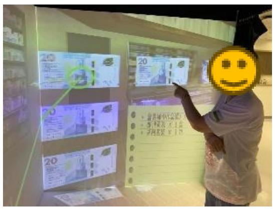 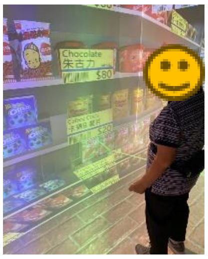

VR Game 3: **Jogging and balancing exercise**

Purpose: Physical balance and safe walking training

Grading: 3 levels (Level 1-by hand trackers; Level 2- by hand trackers & obstacle; Level 3- by hand/foot trackers & obstacles)

Device: 3D stereoscopic eyewear, hand and foot trackers

Jogging in the park<br>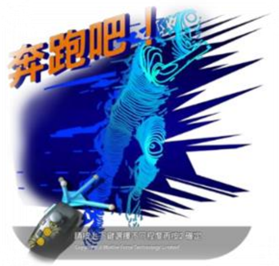<br>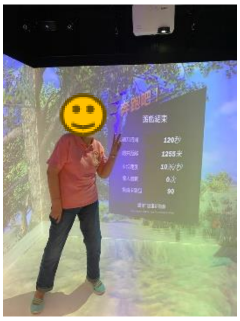	Sequencing & Grading:<br>1. Participant uses hand trackers selects a level of activity<br>2. Participant runs with arms movement and keeps on a running path (upper limb balance)<br>3. Participant runs with upper and lower limbs trackers and follows a running path (whole body balance & physical training)<br>4. Participant runs and avoids hitting any obstacle on a running path (advance physical balance and coordination skill)<br>5. Participant runs faster and overcomes more obstacles (physical and<br>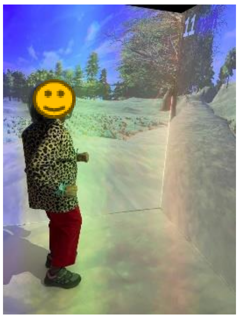 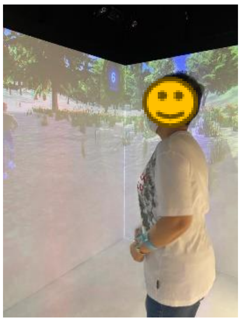 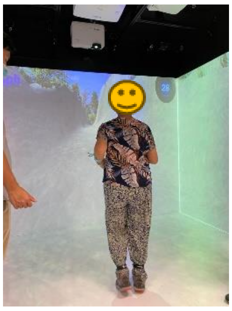<br>cognitive training)

VR Game 4: **Balance & weight-shifting exercise**

Purpose: Cognitive-motor training and advanced balance exercise

Grading: 4 levels:

Level 1- defender (keeping); Level 2-attacker (attacking); Level 3- penalty kick (attacking);

Level 4- penalty kick (goalkeeping)-

Device: 3D stereoscopic eyewear, hand controller, hand, and foot trackers

Soccer exercise	Sequencing & Grading:
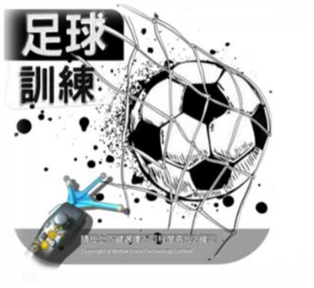	1. Participant uses hand controller selects a level of activity<br>2. Participant wears hand and foot trackers, practices passing a soccer to other player (balance & stability)<br>3. Participant runs and kicks a soccer to different position (balancing & weight shifting training)<br>4. Participant kicks a soccer to goalkeeper repeatedly (balancing, stability, weight-shift training to prevent falling)<br>5. Participant as a goalkeeper uses hand and foot trackers to defense the attacker (advance limbs balancing and coordination, ball tracking exercise, spatial orientation & higher executive function skill)<br>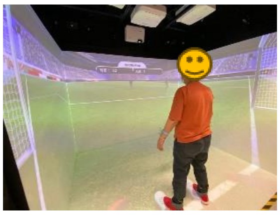 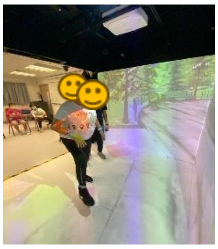

File S3

Figure 2. Change in cognitive function (HK-MoCA), a higher score indicates better cognitive function. VR group showed greater improvement than control group in the study (P value <.005).

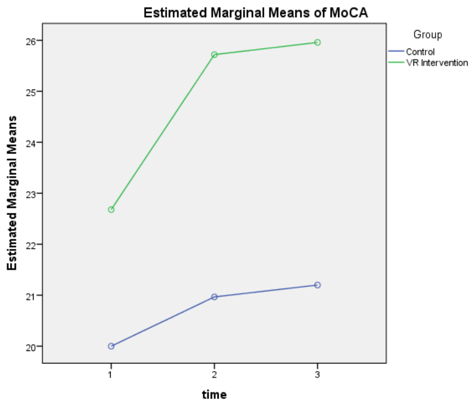

Figure 3. Change in postural balance (Berg Balance Test), a higher score indicates better balance level. VR group showed greater improvement than control group in the study (p value<.05).

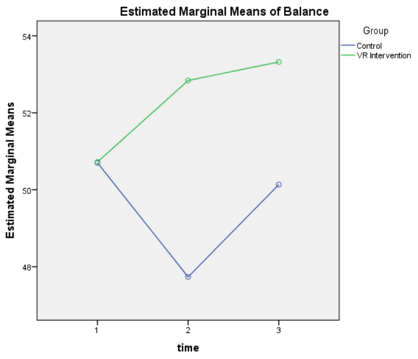

Figure 4. Change in walk speed (6-minute walk test), a longer distance indicates faster walking speed. VR group showed greater improvement than control group (p value <.005).

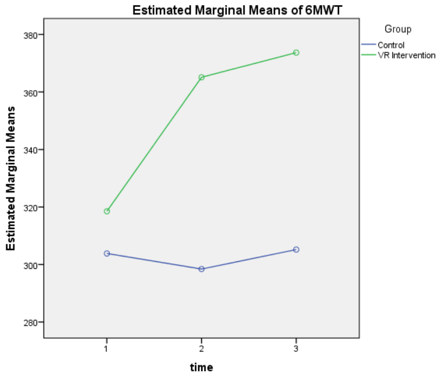

Figure 5. Change in functional mobility (Time Up and Go Test), a shorter time (<12s) indicates better functional level and lesser risk of fall. Two groups showed no significant difference in the study (P value >.05).

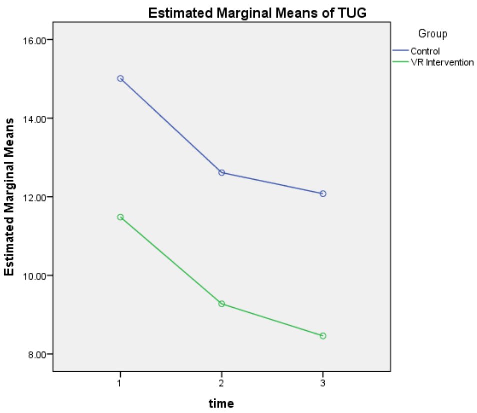

Figure 6. Change in fear of falling (Fall Efficacy Scale), a lessor score (FES-I<28) indicates lower level of fall concern. Two groups showed no significant difference in the study (p value >.05).

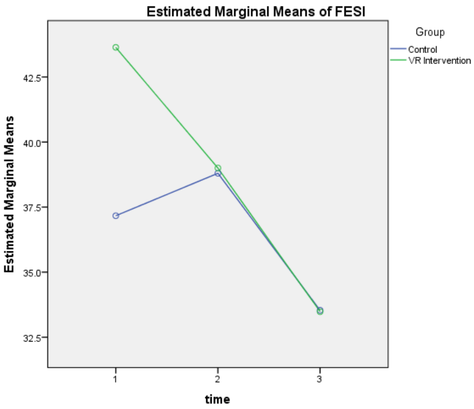

Figure 7 & 8. Change in executive function (Trial Making Test A & B), a shorter time indicates better level of executive function. VR group showed greater improvement than control group in the study (p value <.05).

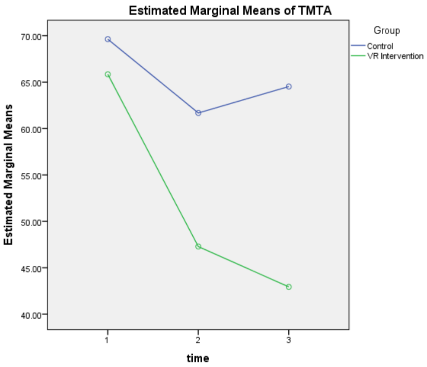

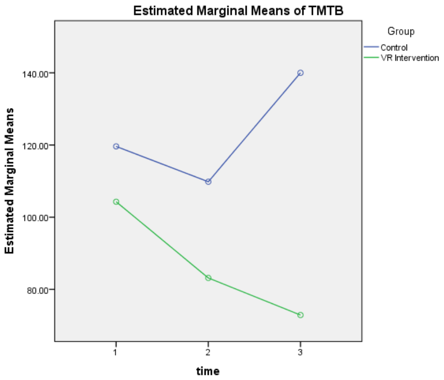

Supplement: Supplementary file 1 [file sensors-25-03123-s001.zip › sensors-3569388-supplementary.pdf]
